# The Silent Period for Small Fiber Sensory Neuropathy Assessment in a Mixed Cohort of Transthyretin-Mediated Amyloidosis

**DOI:** 10.3390/biomedicines10092073

**Published:** 2022-08-24

**Authors:** Chiara Cambieri, Laura Libonati, Federica Moret, Giorgio Tartaglia, Matteo Garibaldi, Cristina Chimenti, Maurizio Inghilleri, Marco Ceccanti

**Affiliations:** 1 Center for Rare Neuromuscular Diseases, Department of Human Neuroscience, Policlinico Umberto I, Sapienza University of Rome, 00185 Rome, Italy; 2 Neuromuscular and Rare Disease Center, Department of Neuroscience, Mental Health and Sensory Organs (NESMOS), Sant’Andrea Hospital, Sapienza University of Rome, 00189 Rome, Italy; 3 Department of Cardiovascular, Respiratory, Nephrologic, Anesthesiologic and Geriatric Sciences, Sapienza University, 00185 Rome, Italy; 4 Cellular and Molecular Cardiology Lab, IRCCS Lazzaro Spallanzani, 00149 Rome, Italy

**Keywords:** transthyretin-mediated amyloidosis, cutaneous silent period, mixed nerve silent period, neurophysiology

## Abstract

Background: Transthyretin-mediated amyloidosis (ATTR) is a rare multisystemic disease involving the peripheral nervous system and heart. Autonomic and small fiber involvement is one of the hallmarks of ATTR, and many tools have been proposed to assess this aspect. Aim: The aim of this study was to investigate cutaneous and mixed nerve silent periods (CSP and MnSP) as instruments for small fiber assessment. Methods: A total of 21 ATTR patients, 20 healthy controls, and 18 asymptomatic carriers underwent a sensory conduction study from the right sural and non-dominant ulnar nerves. A motor conduction study from the right deep peroneal and non-dominant ulnar nerves, with their F waves, CSPs, and MnSPs, was performed. Results: The amplitudes of the sural and ulnar sensory nerves and of the peroneal and ulnar motor nerves were reduced in ATTR patients compared to the other groups. F waves from the ulnar and peroneal nerves showed no differences between the three groups. The CSP and MnSP latency, but not amplitude, were increased in both the ulnar and peroneal nerves of ATTR patients. Conclusions: ATTR patients showed axonal involvement of large sensory and motor nerve fibers and demyelinating features of small sensory fibers.

## 1. Introduction

Transthyretin-mediated amyloidosis (ATTR) is a rare multisystemic disease involving neurologic and cardiovascular systems. Two forms have been described, namely, variant (ATTR-v) and wild-type (ATTR-wt). ATTR-v is associated with mutations of the transthyretin (TTR) gene. In Italy, the disease prevalence is 4.33/million [1], though more recent population studies have reported higher frequencies in some specific regions [2]. In ATTR-wt, the TTR gene has a normal sequence, but the protein precipitates in the elderly.

Among the different tissues and systems involved in amyloidosis, the peripheral neurologic (PNS) and cardiovascular systems are the principal actors, playing a crucial role in prognosis [3]. PNS involvement is characterized by different phenotypes, including sensory, sensorimotor, pure motor axonal, or demyelinating polyneuropathies (ATTR-PN).

A peculiar feature of ATTR-PN is the involvement of small sensory fibers [4] that mediate the autonomic nervous system (ANS). ANS involvement in ATTR-PN has been recognized since early descriptions of the disease [5,6] and gives rise to many specific symptoms, such as postural hypotension, constipation, diarrhea, erectile dysfunction, and others, impacting morbidity, disease progression, and mortality. This fiber set involvement was also observed in ATTR-wt [7,8,9,10].

The ANS targets many different systems, and its evaluation can be challenging. Standard neurophysiologic assessments and nerve conduction studies can only assess large sensory and motor fibers. Other techniques have been proposed to study small sensory fibers, such as Sudoscan [11], laser-evoked potentials [11,12], and skin biopsy [13], though many of these are not widely available.

The cutaneous silent period (CSP) is an advanced neurophysiological tool that reflects the function of A-delta hypomyelinated sensory fibers [14,15,16,17] modulated by the corticospinal tract [18]. The CSP is an inhibitory reflex mediated at the spinal level, with an afferent arc provided by small-diameter A-delta fibers and an efferent arc provided by alpha motoneurons [16,17]. Inhibition of the surface electromyographic trace is obtained by high-intensity and long-lasting stimulation of the cutaneous branches of the sensory nerves during voluntary contraction.

The mixed nerve silent period (MnSP) is another neurophysiologic tool used to assess small fibers [19]. In this case, the inhibition of surface electromyographic activity is obtained through high-intensity and long-lasting stimulation of the nerve trunk. The MnSP is composed of three different parts: the collision of antidromic with orthodromic motor impulses, Renshaw cell inhibition activated by an antidromic motor volley, and the activation of high-threshold cutaneous fibers within the mixed nerve [19]. The latter part has been suggested to share a common inhibitory pool with the CSP [15].

Many trials have used these techniques to assess small fiber neuropathies in diabetes [20,21], chronic inflammatory demyelinating polyneuropathy [20], amyotrophic lateral sclerosis [22], and many other diseases. No paper has ever used these tools to assess A-delta fiber involvement in systemic amyloidosis. CSP and MnSP latencies are related to the myelination state of A-delta fibers, while their duration is related to axon number [20]. In chronic inflammatory demyelinating polyneuropathy, an autoimmune disease with a demyelinating process, CSP latency was increased, while the duration was reduced in axonal polyneuropathies.

## 2. Materials and Methods

### 2.1. Patients

We enrolled 21 patients with ATTR-wt and ATTR-v in this observational study and matched them with 20 healthy controls (HCs) and 18 asymptomatic carriers (ACs) of TTR mutations. Demographic data and physical examinations were collected. None of the patients was prescribed any specific disease-modifying drug for ATTR-PN at the time of the evaluation. Sex matching could not be performed between the three groups, given the high prevalence of males in the symptomatic ATTR population. Nevertheless, previous studies demonstrated no sex differences for the silent period [23].

### 2.2. Neurophysiological Assessment

Each subject underwent a neurophysiological assessment with an evaluation of right sural and non-dominant ulnar sensory nerve conduction and a motor nerve conduction study from the right deep peroneal and non-dominant ulnar nerve. F waves were recorded from the same nerves.

The CSP from the upper limb was obtained by stimulating the fifth non-dominant finger and recording ipsilateral abductor digiti minimi (ADM) surface electromyography during maximal contraction. The CSP was obtained from the lower limb by right dorsal big toe stimulation and recorded from the ipsilateral extensor digitorum brevis (EDB) during maximal contraction.

The MnSP from the upper limb was obtained by non-dominant ulnar nerve stimulation on the wrist and ipsilateral ADM recording during maximal contraction. The MnSP from the lower limb was obtained by peroneal nerve stimulation and ipsilateral EDB recording during maximal contraction.

For both silent periods, the stimulus intensity was 10 times the sensory threshold with a 500 µs duration [24]. A recording time of 1000 ms was analyzed, with a sweep time of 200–300 ms for both the upper and lower limbs. Filters were set as 30–10,000 Hz. Five trials with a frequency of 0.7 Hz were performed for each silent period. Raw traces were rectified and averaged. The silent period latency and duration were calculated at the intersection between 80% of the mean pre-stimulus EMG activity and the averaged trace. For the MnSP, only the late components after the collision wave were considered since this component represents the fibers of interest.

### 2.3. Statistics

All data were analyzed with SPSS Statistics 25.0 (IBM, Chicago, IL, USA). The Chi-squared test was used for nominal variables. Differences between the three groups were tested through non-parametric tests (Kruskal–Wallis) in case of a non-normal distribution. Statistical significance was set as *p* < 0.05.

## 3. Results

### 3.1. Demographic Data

Demographic and neurophysiologic data are reported in Table 1 and Table 2, respectively. No differences in terms of age were found between HCs and ATTR patients, while ACs were significantly younger, as expected (68.2 ± 12.5 vs. 72.7 ± 14.1 vs. 50.1 ± 15.5 years; *p* < 0.05 for AC vs. ATTR and HCs).

### 3.2. Neurophysiological Assessments

The sural and ulnar sensory nerves had reduced amplitude in ATTR patients compared to both HCs and ACs (sural nerve: 3.15 μV ± 2.7 vs. 14.3 μV ± 4.1 vs. 12.5 μV ± 4,7; ulnar nerve: 2.8 μV ± 2.7 vs. 11.3 μV ± 2.2 vs. 11.0 μV ± 2.7; *p* < 0.05 for ATTR vs. ACs and HCs).

The amplitude of the compound muscle action potential (cMAP) from peroneal and ulnar nerves was significantly lower in ATTR patients compared to HCs and ACs (peroneal nerve: 5.2 mV ± 4.0 vs. 9.4 mV ± 3.7 vs. 7.6 mV ± 2.5; ulnar nerve: 11.6 mV ± 5.6 vs. 17.3 mV ± 3.8 vs. 16.4 mV ± 2.7; *p* < 0.05 for ATTR vs. ACs and HCs for both the peroneal and ulnar nerves), while the distal latencies were non-significantly different (peroneal nerve: 3.5 ms ± 0.27 vs. 3.7 ms ± 0.3 vs. 3.5 ms ± 0.29; ulnar nerve: 2.05 ms ± 0.17 vs. 2.1 ms ± 0.14 vs. 2.1 ms ± 0.12).

The CSP and MnSP latencies from the peroneal and ulnar nerves were significantly increased in ATTR patients compared to HCs and ACs (CSP peroneal nerve: 138.2 ms ± 12.3 vs. 108.1 ms ± 21.7 vs. 107.7 ms ± 19.5; MnSP peroneal nerve: 119.2 ms ± 8.0 vs. 98.1 ms ± 15.0 vs. 97.6 ms ± 10.1; CSP ulnar nerve: 92.9 ms ± 13.1 vs. 78.9 ms ± 11.0 vs. 70.5 ms ± 10.2; MnSP ulnar nerve: 77.6 ms ± 9.1 vs. 66.0 ms ± 9.3 vs. 59.2 ms ± 7.9; *p* < 0.05 for ATTR vs. ACs and HCs for both the peroneal and ulnar CSP and MnSP latencies), while the duration was the same, (*p* > 0.05), even if a trend to significance was found in the peroneal CSP and MnSP duration of ATTR patients compared to HCs and ACs (CSP peroneal nerve: 43.5 ms ± 8.4 vs. 56.9 ms ± 17.9 vs. 68.5 ms ± 27.5; MnSP peroneal nerve: 53.4 ms ± 23.8 vs. 63.6 ms ± 11.9 vs. 67.2 ms ± 19.0).

**Figure 1:**
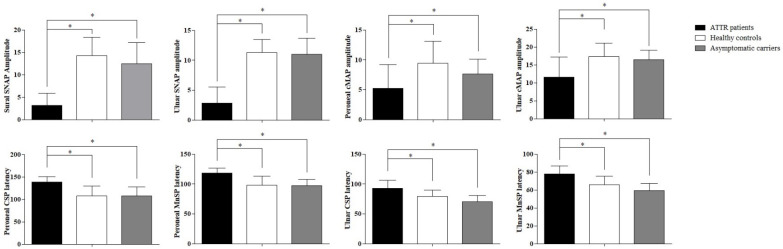
Neurophysiologic differences between the three groups. * *p* < 0.05.

No differences were found in terms of the F wave latencies of the peroneal and ulnar nerves between ATTR patients, HCs, and ACs (peroneal nerve: 49.6 ms ± 7.7 vs. 48.2 ms ± 4.8 vs. 47.5 ms ± 3.3; ulnar nerve: 28.8 ms ± 9.6 vs. 27.7 ms ± 2.6 vs. 27.0 ms ± 2.4; *p* > 0.05 for both the peroneal and ulnar nerves). All the data are illustrated in Figure 1.

No differences were found in any neurophysiological test between HCs and ACs.

## 4. Discussion

ATTR is a systemic disease involving many organs and systems. Within the nervous system, both the central and peripheral districts may be involved, although the involvement of the latter is more common. The PNS has a complex structure, with many different fibers providing motor and sensory functions. Regarding sensory functions, the A-delta and small C fibers mediate sensitivity to pain and temperature. Historically, ATTR was considered a systemic disease involving small sensory fibers and leading to painful polyneuropathies. Small fiber neuropathy accounts for some of the most typical signs in ATTR [25,26,27], including painful neuropathy [28], orthostatic hypotension [29], erectile dysfunction [30,31,32,33], constipation or diarrhea [34], and heart autonomic dysfunction [35,36,37].

Although electrophysiology is considered the gold standard for the diagnosis and follow-up of large sensory and motor fiber involvement, many different tests are applied with the same aim for small fibers: the composite autonomic symptom score (COMPASS) and its abbreviated form (COMPASS 31) [38,39], quantitative sensory testing [40]; the sympathetic skin response [41], laser-evoked potentials (LEPs) [42,43], and Sudoscan [44,45]. Nowadays, skin biopsy is considered the gold standard for the diagnosis of small fiber neuropathy [46] due to its high sensitivity and ability to assess unmyelinated C fibers, though its mild but significant invasiveness precludes its use for follow-up in a clinical setting.

All of these tools have strengths and weaknesses that may limit their clinical use in terms of reproducibility, invasiveness, sensitivity, specificity, and accuracy; some of them can evaluate only the symptomatic patients. Finally, many of these tests are not widely available.

To resolve many of these issues, we propose the CSP and MnSP as reliable, reproducible tests to evaluate A-delta sensory fibers in ATTR patients. The CSP and MnSP are well-characterized neurophysiological tests that represent protective reflexes mediated by spinal inhibitory circuits [15,16,17]. They are obtained with supramaximal stimulation of the skin/nerve during voluntary muscle contraction. Many authors have defined their physiological circuit, which involves A-delta small-diameter sensory fibers [14,15,47]. In a previous trial, the CSP was shown to have increased latency and reduced duration in demyelinating and axonal polyneuropathies, respectively [20].

In this study, we used the CSP and MnSP to further describe the involvement of small fibers in ATTR patients. We used these tools to evaluate autonomic dysfunction and diagnose the involvement of this set of fibers. We found axonal loss in sensory and motor fibers in ATTR patients compared to HCs and ACs, as demonstrated by the reduced cMAP amplitude of the peroneal and ulnar motor nerves. Although demyelinating features have sometimes been described by other authors [48], no patient in our cohort met the new European Federation of Neurological Societies/Peripheral Nerve Society (EFNS/PNS) criteria for demyelinating polyneuropathy [49], at least in terms of F wave latency and distal motor latency. Indeed, reduced nerve conduction velocity in ATTR patients is usually associated with a marked reduction in motor and sensory action potential amplitudes [48]. However, the EFNS/PNS criteria evaluate the demyelinating process on large sensory and motor fibers only and not the myelination state of A-delta hypomyelinated fibers.

In this observational study, the CSP and MnSP latencies from the peroneal and ulnar nerves were increased in ATTR patients compared to HCs and ACs, while the duration was preserved. These neurophysiological findings suggest a demyelinating process affecting the A-delta fibers. A non-significant reduction in the CSP and MnSP duration was found in the peroneal nerve within the ATTR group compared to HCs and ACs, suggesting a possible early A-delta axonal loss in the lower limbs. A reflex arc mediates the CSP and MnSP, with the A-delta small fibers as the afferent and alpha motoneurons as the efferent branch. The F wave and the distal motor latency were tested in ATTR patients, HCs, and ACs to rule out the effect of possible alpha motoneuron demyelination in increasing CSP and MnSP latencies. The similar F wave latency and distal motor latency confirmed sensory A-delta fiber demyelination. A possible limitation of the study is the lack of proximal site motor nerve stimulation; nevertheless, the F wave latency was abundantly within the normal ranges from both the peroneal and ulnar nerves, thus suggesting a normal myelination of the whole motor nerve length. Moreover, the proximal nerve site, only investigable through the F wave, is expected to be involved early in ATTR-PN, as discussed below.

The pathophysiological neurodegenerative process affecting the PNS in ATTR has been well-characterized in the past years. In particular, the dorsal root ganglia, sensory and motor roots, and autonomic ganglia, where the blood–nerve barrier is more permeable [50,51], were demonstrated to be the initial sites of amyloid deposition, followed by proximo-distal gradient spreading [52,53]. Among many hypothesized neurodegenerative mechanisms, such as toxic damage [54] or nerve ischemia caused by perivascular amyloids [55], mechanical compression of the nerve fibers in the dorsal root ganglia, autonomic ganglia, and dorsal and ventral roots was demonstrated, which distorted the myelin sheath, causing segmental demyelination and secondary axonal degeneration (Wallerian degeneration) [50]. Different grades of sensory fiber involvement were demonstrated between early- and late-onset ATTR-v patients, with the latter showing prevalent axonal loss of the large fibers and partial axonal sparing of the hypo- and unmyelinated sensory fibers [56,57]. Nevertheless, autonomic and small fiber neuropathies can also be found in late-onset Val30Met as well as in Val30Met ATTR-v and ATTR-wt patients.

This observational study was set in a non-endemic country and enrolled late-onset patients. Axonal loss in large sensory fibers and alpha motoneurons was confirmed in this cohort of ATTR patients, while in small sensory A-delta fibers, we found an initial demyelinating process with no axonal loss or Wallerian degeneration at the time of the examination. On the contrary, the axonal loss evidenced in large motor and sensory fibers suggested early Wallerian degeneration in these fiber sets. According to the hypothesis of an early root demyelination, starting where the blood–nerve barrier is more permeable, we can suppose a proximal demyelination in A-delta fibers when a Wallerian degeneration has already affected the large sensory and motor fibers.

Unfortunately, no early signs of A-delta demyelination were demonstrated by the CSP and MnSP in AC, thus discouraging the use of these tools as early markers of ATTR-PN. Nevertheless, this is the first study demonstrating the role of the CSP and MnSP in symptomatic ATTR. However, the role of these instruments may not just be limited to the study of the pathophysiological mechanisms of PNS involvement in ATTR, but due to their reproducibility, these tools may also be proposed for the systematic assessment of ACs close to the PADO (predicted age of disease onset) or follow-up in treated patients to explore small A-delta fiber involvement. Other studies are necessary to further describe the possible axonal involvement of A-delta fibers in more advanced disease stages and to relate the neurophysiologic data with dysautonomic scales. Further studies should also investigate the different involvement of the silent period in ATTR-v vs. ATTR-wt and in the different disease phenotypes (pure cardiologic vs. pure neurologic vs. mixed phenotype).

## 5. Conclusions

The CSP and MnSP can be proposed as add-on tools to objectively explore small fiber involvement and to distinguish asymptomatic and symptomatic patients, thus applying new instruments to assess the time of disease onset in ATTR-v.

## Figures and Tables

**Table 1:** Demographic data from the three participant cohorts. n.a.: not applicable.

	ATTR Patients	Healthy Controls (HCs)	Asymptomatic Carriers (ACs)	*p*-Value < 0.05
Age (years)	72.7 ± 14.1	68.2 ± 12.5	50.1 ± 15.5	ATTR vs. HCs (*p* < 0.001) and ACs (*p* = 0.002)
M/F	16/5	11/9	6/12	ATTR vs. HCs (*p* = 0.007) and ACs (*p* = 0.007)
ATTR-wt/variant	16/5	n.a.	n.a.	
Pathologic variants	2 Phe64Leu 2 Val30Met 1 Val122Ile	n.a.	3 Phe64Leu 13 Val30Met 2 Val122Ile	
Phenotype: Pure cardiologic	2	n.a.	n.a.	
Pure neurologic	4	n.a.	n.a.	
Mixed phenotype	15	n.a.	n.a.	

**Table 2:** Neurophysiologic data from the three participant cohorts.

	ATTR Patients	Healthy Controls (HCs)	Asymptomatic Carriers (ACs)	*p*-Value < 0.05
Sural SNAP amplitude (μV)	3.15 ± 2.7	14.3 ± 4.1	12.5 ± 4.7	ATTR vs. HCs (*p* < 0.001) and ACs (*p* < 0.001)
Ulnar SNAP amplitude (μV)	2.8 ± 2.7	11.3 ± 2.2	11.0 ± 2.7	ATTR vs. HCs (*p* < 0.001) and ACs (*p* < 0.001)
Peroneal				
cMAP amplitude (mV)	5.2 ± 4.0	9.4 ± 3.7	7.6 ± 2.5	ATTR vs. HCs (*p* = 0.026) and ACs (*p* = 0.037)
cMAP latency (ms)	3.5 ± 0.27	3.7 ± 0.3	3.5 ± 0.29	
F-wave latency (ms)	49.6 ± 7.7	48.2 ± 4.8	47.5 ± 3.3	
CSP latency (ms)	138.2 ± 12.3	108.1 ± 21.7	107.7 ± 19.5	ATTR vs. HCs (*p* = 0.006) and ACs (*p* = 0.019)
CSP duration (ms)	43.5 ± 8.4	56.9 ± 17.9	68.5 ± 27.5	
MnSP latency (ms)	119.2 ± 8.0	98.1 ± 15.0	97.6 ± 10.1	ATTR vs. HCs (*p* = 0.005) and ACs (*p* = 0.028)
MnSP duration (ms)	53.4 ± 23.8	63.6 ± 11.9	67.2 ± 19.0	
Ulnar				
cMAP amplitude (mV)	11.6 ± 5.6	17.3 ± 3.8	16.4 ± 2.7	ATTR vs. HCs (*p* = 0.003) and ACs (*p* = 0.042)
cMAP latency (ms)	2.0 ± 0.17	2.1 ± 0.14	2.1 ± 0.12	
F-wave latency (ms)	28.8 ± 9.6	27.7 ± 2.6	27.0 ± 2.4	
CSP latency (ms)	92.9 ± 13.1	78.9 ± 11.0	70.5 ± 10.2	ATTR vs. HCs (*p* = 0.008) and ACs (*p* = 0.002)
CSP duration (ms)	51.2 ± 23.5	54.2 ± 13.4	53.9 ± 23.5	
MnSP latency (ms)	77.6 ± 9.1	66.0 ± 9.3	59.2 ± 7.9	ATTR vs. HCs (*p* = 0.007) and ACs (*p* = 0.002)
MnSP duration (ms)	56.2 ± 14.1	54.8 ± 16.0	63.4 ± 35.2	

*SNAP*: sensory nerve action potential; *cMAP*: compound muscle action potential; *CSP*: cutaneous silent period; *MnSP*: mixed nerve silent period.

## Data Availability

Not applicable.
